# The use of digital technologies by people with mild-to-moderate dementia during the COVID-19 pandemic: A positive technology perspective

**DOI:** 10.1177/14713012221079477

**Published:** 2022-03-25

**Authors:** Catherine V Talbot, Pam Briggs

**Affiliations:** Department of Psychology, 276175 Bournemouth University, Poole, UK; Department of Psychology, 5995 Northumbria University, Newcastle upon Tyne, UK

**Keywords:** Alzheimer, social media, online communities, social inclusion, support

## Abstract

A growing body of research has shown that people with dementia are using digital technologies to enhance lived experience. The COVID-19 pandemic has brought new digital opportunities and challenges and so provides a unique opportunity to understand how people with dementia have adapted to this new digital landscape. Semi-structured interviews were conducted with 19 people with dementia and analysed thematically. We generated five themes, showing how participants used digital means to combat the stresses of the pandemic by facilitating social connection, self-actualisation, enhanced well-being and by assisting with activities of daily life. These technologies helped to reduce isolation, provide access to support groups, create opportunities for cognitive stimulation and self-development, and engendered a sense of identity at a time of perceived loss. Despite these benefits, participants also reported challenges regarding cognitive fatigue and usability issues. We recommend that training on how to use digital technologies is co-produced with people with dementia and designers engage with the voices of people with dementia throughout the design process. In turn, this could promote the social connectedness, well-being and self-worth of people with dementia.

## Introduction

There are an estimated 54 million people living with dementia worldwide, with numbers set to rise to 82 million by 2030 (World Health Organization, 2020). Dementia is a syndrome characterised by progressive cognitive decline and functional impairments that impact daily life. In the mild to moderate stages, each type of dementia has a different profile – although as dementia progresses these differences become less distinguishable. For example, the early stages of Alzheimer’s disease can be characterised by impairments in episodic memory, whereas altered social comportment may be more pronounced in frontotemporal dementia and visuospatial deficits more severely impaired in dementia with Lewy bodies (Weintraub, Wicklund, & Salmon, 2012). These cognitive changes are associated with declining functional ability (Royall et al., 2007; Martyr & Clare, 2012). During the early stages of dementia, instrumental activities of daily living show noticeable decline, such as using a telephone, preparing meals, performing household tasks, shopping for food and handling finances or medication (Pérès et al., 2008). In turn, these impairments can impact upon independence, confidence and a person’s ability to ‘live well’ (Martyr et al., 2019).

A diagnosis of dementia can also have a powerful social impact on a person, negatively affecting their sense of identity, self-worth and self-esteem (Roach & Drummond, 2014; Spreadbury & Kipps, 2019; Talbot et al., 2021); enhancing feelings of isolation, uncertainty and frustration (Beard & Fox, 2008; Langdon, Eagle, & Warner, 2007); reducing meaningful social roles and diminishing power in social relationships (Ryan, Bannister, & Anas, 2009). Others have theorised that people with dementia can experience a ‘shrinking world’, referring to a reduction in the number of places in which they feel comfortable (Duggan et al., 2008; Talbot & Briggs, 2021). People with dementia also face significant stigma, whereby those with the diagnosis are often identified as ‘victims’ and ‘sufferers’ (Peel, 2014) and their symptoms portrayed negatively and unrealistically (Gerritsen, Kuin, & Nijboer, 2014).

With recent medical and policy initiatives focused on diagnosing people in the early stages of dementia (e.g. Department of Health, 2016), it has become increasingly important to support those with the diagnosis to ‘live well’. By facilitating social connection and self-expression, digital technologies may provide one way of combatting the challenges posed by the diagnosis. For example, people with dementia have been found to use social media (Kannaley et al., 2019; Talbot et al., 2020a, 2020b, 2021; Thomas, 2017), emails and online chat rooms (Clare, Rowlands, & Quin, 2008), smartphones and tablet devices (Lim et al., 2013; Joddrell & Astell, 2016). These technologies can be beneficial as they facilitate peer support and social interaction, and can engender a sense of identity at a time of perceived loss (Clare et al., 2008; Craig & Strivens, 2016; Kannaley et al., 2019; Talbot et al., 2020b, 2021). However, human–computer interaction (HCI) research has traditionally focused on protecting or monitoring people with dementia (Astell, 2006; Kerssens et al., 2015), and digital technologies often do not consider the cognitive and sensory changes that the person may be experiencing (Joddrell & Astell, 2016; Smith & Mountain, 2012). Such approaches may be inadvertently paternalistic and ineffective as they do not consider the concerns of people with dementia or view them as human beings who experience the world.

More recently, HCI researchers have adopted an alternative approach that champions personhood and identifies dementia as a social construction (Lazar, Edasis, & Piper, 2017; Madjaroff & Mentis, 2017; Dixon & Lazar, 2020). Lazar et al. (2017) coined the term ‘critical dementia’ to refer to this position, taking a broad view that considers context, embodiment, sensorial experiences and emotion. Rather than taking a purely cognitive view of the mind and viewing computers as simply tools for work, HCI now embraces the emotional, embodied and cultural aspects of interactions with technologies in everyday life (Lazar et al., 2017). A critical dementia view therefore moves away from solely viewing dementia in terms of loss and disability, but instead recognises the needs of the person and explores how to support their potentiality, abilities, identities and sense of self (Brankaert et al., 2019). We adopt this stance in our work, in which we recognise the situatedness of technology use and value the emotional experiences of people with dementia. Thus, we recognise the importance of the social environment in shaping experience and view people with dementia as active citizens (see Bartlett, 2016; Baldwin, & Bradford Dementia Group, 2008).

There is a greater need to understand the ways that digital technologies may enhance the lived experience of people with dementia (Neal et al., 2021) and this has become more pressing during the COVID-19 pandemic since digitally mediated interactions have become a necessity for many (Beaunoyer, Dupéré, & Guitton, 2020). Research that explores the ways that people with dementia have taken up new technologies and platforms during this time will yield further insights into the benefits and challenges for this population. This will be vital in informing care, services, policymaking and the development of digital technologies and interventions that aim to support people with dementia.

During the COVID-19 pandemic, people with dementia were at risk of negative outcomes. They were adversely affected by a disruption of routines, lack of cognitive stimulation, reduced social interactions, perceived symptom progression and a rapidly ‘shrinking world’ (Giebel et al., 2020; Talbot & Briggs, 2021). One key concern related to the social connectedness of people with dementia, with the Alzheimer’s Society (2020) reporting surges in loneliness and isolation among this group – factors associated with a person’s ability to ‘live well’ (Clare et al., 2019). Restricted access to support groups and services may have contributed to this increase (Giebel et al., 2020; Talbot & Briggs, 2021), given that these have been found to be effective in combatting loneliness and isolation (Giebel, Chalis, & Montaldi, 2016; Willis, Semple, & Waal, 2018). The potential of digital technologies to foster social connection beyond direct networks may provide a valuable tool for people with dementia to combat the isolation that often accompanies lockdown periods and the diagnosis itself (Spreadbury & Kipps, 2019; Talbot & Briggs, 2021).

Here, we adopt a ‘positive technology’ lens to investigate digital technology usage by people with dementia, where positive technology refers to the scientific and applied approach to the use of technology for enhancing personal experience (Gaggioli et al., 2019; Wiederhold & Riva, 2012). Riva, Montovani and Wiederhold (2020) argued that positive technologies can complement existing strategies for generating well-being, identifying three types of technology that can enhance experience: (1) ‘Hedonic technologies’ that induce positive emotional states; (2) ‘Eudemonic technologies’ that support individuals in reaching, engaging and self-actualising experiences and (3) ‘Social/interpersonal technologies’ that promote integration and connectedness between individuals, groups and organisations. Positive technologies have typically been virtual reality environments and other forms of software design as interventions for mental health and well-being (Peters, Calvo, & Ryan, 2018). However, everyday digital technologies may also enhance personal experience, particularly during the pandemic when opportunities for social interaction and self-actualisation were limited. For example, video game play has been associated with positive emotional states (Johannes, Vuorre, & Przybylski, 2021); conducting volunteer work via telephone or video-calling software can facilitate purpose (Son et al., 2020); social media can foster a sense of social connectedness (Sun, Rieble, Liu, & Sauter, 2020). Such digital technologies may be equally valuable for people with dementia, yet to our knowledge no researchers have used a positive technology lens to investigate this.

Despite the potential of digital technologies, we recognise that some people with dementia may have faced challenges, particularly relating to access and digital literacy. For example, in work that examined experiences of accessing post-diagnostic dementia care, respondents found online forms of support to be unsuitable and inaccessible for those without access to the internet and electronic devices (Giebel et al., 2021). The symptoms of dementia may also create difficulties when using digital technologies. Giebel et al. (2020) argued that people with visual types of dementia (e.g. posterior cortical atrophy) in particular may struggle to use technologies and, consequently, find it difficult to access remote services. Consistent with this, people with dementia have reported challenges using social media, highlighting that these platforms require considerable cognitive effort (Talbot et al., 2021). Moreover, research suggests that people with dementia may encounter social challenges online, such as encountering stigmatising language and negative comments (Oscar et al., 2017; Talbot et al., 2021). Exposure to such comments may impact their well-being and sense of identity and serve to deter them from online spaces.

The design of digital technologies may also pose challenges for people with dementia. Joddrell and Astell (2016) provided a comprehensive overview of relevant design issues, which includes visual design, feedback, screen size, customisation and intuitive control. That is not to say that people with dementia are incapable of using digital technologies, rather that technologies have been designed in a ‘hypercognitive society’ (Post, 2000) which makes implicit assumptions about a person’s cognition. Thus, the designs of digital technologies may not be accessible for people with dementia, thereby inadvertently contributing to their social exclusion.

It is also possible that the dominance of digital technologies during the COVID-19 pandemic may have exacerbated already existing inequalities and a ‘digital divide’, not only for people with dementia but also other groups without ready access to the internet or sufficient digital literacy skills (Lai & Widmar, 2020). Given that there is growing pressure for offline spaces to be dementia-friendly (e.g. Alzheimer’s Society, 2013), it is essential that researchers identify ways in which digital spaces can also be accessible for people with dementia. It is therefore important to examine what challenges people with dementia face when using digital technologies so that future adaptations can be made to promote their digital inclusion.

In this research, we use the COVID-19 pandemic as a case when digital technology usage was enhanced. We ask, ‘how and why did people with mild-to-moderate dementia use digital technologies during the COVID-19 pandemic?’ to garner insights into the benefits and challenges that these technologies confer.

## Method

### Design

This analysis is part of a wider qualitative study that examines the impact of the pandemic on people with dementia (Talbot & Briggs, 2021). In this phase of the study, we report an analysis of interviews conducted with people with dementia and specifically focus on their use of digital technologies.

### Sample

The study was advertised on Twitter and subsequently shared by the accounts of dementia organisations and networks. The study was also advertised on dementia organisations and networks’ websites and in Facebook groups for people with dementia, after receiving permission from group moderators. Study details were also shared with facilitators of peer support and advocacy groups, who subsequently shared the study with group members. Participants were included in this study if they: (1) self-identified as a person with dementia; (2) lived in the United Kingdom and (3) had capacity to give informed consent.

Nineteen participants were recruited, with seven identifying as female and 12 as male. Participants were relatively young, with a mean age of 62.47 years (range: 50–84 years, *SD* = 7.05 years) and 14 having young-onset dementia (i.e. diagnosed before the age of 65). All participants were living with mild-to-moderate dementia. Fourteen participants lived with family members and five lived independently; no participants lived in a care home. All participants reported being users of technology; however, some participants only started using certain types of technology (e.g. social media and video-calling software) during the pandemic. Table 1 provides demographic information about the sample.

**Table 1.** Participant information.

ID	Gender	Age	Living situation	Type of dementia
P1	Female	58	Living with family	Alzheimer’s disease
P2	Male	59	Living with family	Mixed
P3	Male	84	Living with family	Alzheimer’s disease
P4	Female	55	Living with family	Alzheimer’s disease
P5	Male	57	Living with family	Mixed
P6	Male	61	Living with family	Vascular
P7	Female	62	Living with family	Alzheimer’s disease
P8	Male	62	Living with family	PCA^a^
P9	Male	63	Living with family	Frontotemporal
P10	Male	62	Living independently	PCA^a^
P11	Male	68	Living independently	Alzheimer’s disease
P12	Male	55	Living with family	Alzheimer’s disease
P13	Female	64	Living with family	Vascular
P14	Male	62	Living with family	Vascular
P15	Male	68	Living with family	Vascular
P16	Male	67	Living with family	Alzheimer’s disease
P17	Female	66	Living independently	Mixed
P18	Female	50	Living independently	PCA^a^
P19	Female	64	Living independently	Mixed

^a^Posterior cortical atrophy.

### Procedure

After obtaining consent and checking capacity, the first author conducted semi-structured interviews with participants. Interviews took place between June and July 2020, as restrictions first started to ease in the United Kingdom. Interviews were conducted remotely because of social distancing measures. Nine interviews were conducted using video-calling software, nine by telephone, and one by email. The choice of platform was dependent on participant’s preferences and access to/familiarity with technology. The interviews were guided by an interview schedule, which explored participants’ experiences of the pandemic and their use of technology. Interviews lasted between 30–60 min and were recorded using an encrypted digital Dictaphone. Interview data were transcribed verbatim and anonymised.

### Analysis

Interview data were analysed thematically, following Braun and Clarke’s (2006) approach. The first author began by familiarising herself with the data by reading and rereading interview transcripts, noting initial coding ideas for the subsequent phases of the analysis. The data were then coded semantically, reflecting the explicit content of the data. Coding was then reviewed by the second author who provided suggestions on how to interpret the data. Theme development was led by the first author and the second author provided consultation. The development of themes was guided by a positive technology lens (Riva et al., 2020), where we focused on the reasons why participants turned to digital technologies and how these technologies enhanced personal experience. However, we were also alert and sensitive to the potential challenges people with dementia could face (e.g. ‘Zoom fatigue’, digital inclusion). The analysis was an iterative and reflective process, whereby the authors repeatedly returned to the data and literature until both authors agreed on the final themes.

### Ethics

Ethical approval was obtained from the host institution (ID: 24532). In accordance with the Mental Capacity Act (Department of Health, 2005), participants were assumed to have capacity unless they demonstrated otherwise. Capacity was assessed remotely at the start of each interview. A checklist was used to determine participants’ ability to understand and retain information about the research, to weigh up that information to reach a decision and to state a decision clearly. Informed consent was also obtained from participants’ carers and participants were made aware that a carer or family member could also attend the interview if they wished. Consent was viewed as a dynamic and evolving process, whereby the researcher checked that participants were happy to continue with the study throughout the interview. If there was any doubt about capacity, consent was not taken. In this research, however, all participants demonstrated that they had capacity.

## Results

We generated five themes: (1) Technologies to assist with everyday life; (2) Technologies for social connection; (3) Technologies to feel good; (4) Technologies to find meaning; and (5) Hypercognitive technologies. Participants generally reported positive experiences of using digital technologies, which may be because all participants reported using technologies to some degree. Our themes speak to the adaptability of participants, who used digital technologies to counter the stresses of the pandemic, demonstrating agency and a willingness to use digital technologies.

### Technologies to assist with everyday life

Participants described the ways in which they used digital technologies to support everyday life. During lockdown, people were encouraged to remain home, leaving the house simply for exercise. This meant that established routines were disrupted and for some, the outdoors became increasingly unfamiliar (see Talbot & Briggs, 2021). Some participants described using digital tools such as Google Maps more frequently during the pandemic, using it to orient themselves when leaving the house. In part, participants attributed this change to perceived symptom progression. For example, P10 recalled his experience of suddenly feeling lost on a local walk, but using Google Maps and photographs of landmarks on his phone to find his way again:

Every Sunday I’d go for a stroll. It is it’s very nice, and I go for a walk most mornings early doors. But I started losing track of where I am. And it’s weird. So now what I’ve done is I’ve just used Google maps and I do photographs of landmarks and keep them on my phone and that’s okay. (P10)

By using technology in this way, P10 was able to continue engaging with the outdoors and maintain his independence. Another key way in which people with dementia used digital technologies was for online shopping, primarily food shopping. Many of these participants turned to online shopping because they felt unable to visit stores offline due to a lack of dementia-friendly practices, practical issues with shopping and concerns about contracting COVID-19.

I’m keeping away from shops because I’m not sure exactly how the system in the shops are working anymore I’m just used to going into a shop, getting what I want, and getting out. There seems to be a system now so that’s very very difficult to try and get to grips with. (P2)

While some participants had success with online shopping, many experienced challenges as they were unable to obtain a delivery slot. Consequently, participants reported feeling anxious about whether they would be able to obtain groceries and often became reliant on the good will of family members, neighbours and others in the local community:

We were helped by nobody, and if we didn’t have family to help us, we would be struggling. We tried to do online shopping and it was like a three week wait. You can’t do without food for three weeks. (P6)

As I say I’ve been lucky because I’ve had my village shop because I couldn’t get online shopping. (P17)

This is consistent with other research conducted with people with dementia and unpaid carers, who also reported struggles purchasing groceries during the pandemic (e.g. Giebel et al., 2021). In part, difficulties obtaining delivery slots appeared to be the result of people with dementia not being classed as a vulnerable group, with many participants feeling ‘forgotten’ or ‘side-lined’:

We couldn’t get on to do the online shopping because there was no spaces left, you know I’ve had to rely on hubby because family don’t live nearby. There was nothing set up for any help for shopping. I don’t think they looked at the people with dementia as being vulnerable. (P4)

It would have helped me if I’d have had a priority online shopping. So, it’s simply the recognition that we exist that wasn’t done at the beginning. (P17)

These findings suggest that while digital technologies for online shopping may be beneficial in theory, they are only effective in supporting those most vulnerable in society when policymaking is effective. Like Giebel et al. (2021), our findings highlight practical access issues, which emphasise the need for increased recognition of the needs of people with dementia and support for equitable access to basic essentials.

### Technologies for social connection

Participants reported enhanced levels of loneliness and isolation during the pandemic, reflecting prior work (Alzheimer’s Society, 2020; Giebel et al., 2020). To combat these feelings, participants turned to video-calling software and social media to connect with their peers and access much needed support:

It was hard. It was, to see it day after day. It’s the same old every day. It’s a good job I have people to talk to online, because it could have been a lot worse. (P14)

Being able to connect with others with dementia was a ‘lifeline’ for some participants. They described using social media and video-calling software to share strategies on how to cope with the stresses of the pandemic, highlighting the importance of speaking to people who faced similar difficulties:

I think on a bad day when you can’t function and can’t think straight, just having someone outside the home and someone who’s got knowledge of dementia is vital really. (P7)

Being able to connect with people outside the home environment was important for participants, who often felt they could not speak openly with family members. For example, P1 said she could not speak candidly about dementia with family members but could speak freely with her peers online:

It’s not like talking with other people who have got it. You laugh, you joke, you can laugh at yourself. My husband’s still coming to terms with it, and I can’t make silly jokes to make myself feel better because it’s not fair to him. It was very difficult but what they did with the [dementia group], we still have a meeting once a month but on Zoom, so that was lovely. (P1)

These statements suggest that participants felt a sense of community among other people with dementia; as P10 stated: ‘We used to be united against dementia, now we’re united against COVID-19’. Here, we are reminded of the value of group membership, which can provide people with the social and psychological resources to counter challenges of everyday life (Haslam et al., 2009) and may also be beneficial in digital settings (Stuart et al., 2022). We interpret participants’ statements as evidence for digital technologies engendering a collective sense of identity, which enhanced well-being and provided a protective buffer against the isolation that accompanied lockdown measures.

Some participants experienced difficulties when using digital technologies to connect with others during the pandemic. To maintain their sense of social connectedness, they reported modifying the ways in which they used technology, exemplifying a willingness to continue using it. For example, P12 described his experience of using a speech-to-text application to facilitate communication with others:

That’s a pretty good bit of technology for me because I just found it so difficult and long-winded to write, that I wasn’t communicating, but now that I’ve got this app. I’ve got it on my phone and laptop. I can still do the odd emails, which is quite good because it’s great to have everybody to help you, but it’s still nice to do stuff yourself. (P12)

For P12, a speech-to-text app was important, given that opportunities for communication with people outside of the home environment was limited. These technologies appeared to not only improve the social connectedness of participants but also promote their independence by reducing the need for them to rely on others.

Interestingly, some participants felt that videoconferencing software made dementia groups more accessible as it removed the need for travel. As dementia groups moved online, participants reported attending more group meetings; in turn, this expanded their social networks. For example, P19 described her experience of meeting peers weekly (rather than monthly) and connecting with people from different places:

We used to meet every month but during this, we’ve met every week online, which has meant that some people that couldn’t get there because they were just too far away sometimes could join us! It opened new opportunities. (P19)

These findings suggest that technologies such as videoconferencing software provide opportunities for social connection outside of one’s direct offline networks. Therefore, these technologies may provide one way of countering the ‘shrinking world’ that often accompanies dementia (Duggan et al., 2008) and has been accelerated by the pandemic (Talbot & Briggs, 2021).

Despite these benefits, participants also discussed the limitations of virtual peer support. While participants said they felt socially connected during group calls, they missed physical expressions of support, such as a hug, handshake or pat on the back:

Even though I have Zoom, it’s the people contact, the hugs, shaking people’s hands, seeing somebody you know face to face because we just haven’t had that. (P4)

While our findings suggest that interpersonal technologies provided an important coping mechanism for people with dementia during the COVID-19 pandemic, participants’ statements indicate that it was not a direct substitute for in-person support and may not be a suitable long-term means of support.

### Technologies to feel good

Participants described using technologies to enhance well-being, consistent with Riva et al.’s (2020) framework. Participants explained that they struggled with boredom during the pandemic due to having more time available and, consequently, used technologies for enjoyment and to keep themselves occupied. This included using videoconferencing software to take part in activities, such as Pilates, aerobics, deep-breathing exercises and birdwatching, and to view theatre performances:

Monday, we have the local councillor come along and we have someone doing chair-obics for half an hour. On a Tuesday is breathing exercises and then there’s an extra hour afterwards for chair-obics. (P5)

Another way in which participants used technology to enhance well-being included using it for reminiscence, which has been found to have positive short-term effects on cognition, quality of life, communication and mood (O’Philbin et al., 2018). Participants reported taking part in group video calls, in which they discussed photos or music that was important to them:

We said pick three songs and say why they were very important to you. Everyone did that and it almost brought you to a time of your life with a story. There’s a story for every song you know, it’s great. (P16)

These findings suggest that reminiscence can be facilitated by digital technologies and was an important coping strategy for some, transporting them to happier times that allowed them to forget about the stresses of the pandemic.

Previous work suggests that a lack of cognitive stimulation during the pandemic potentially accelerated symptom progression among people with dementia (Alzheimer’s Society, 2020; Giebel et al., 2020). When reflecting on their use of technology, participants often described their usage in terms of keeping their brain ‘ticking over’. For example, P16 felt that taking part in Twitter chats and video calls kept his brain active:

It would have got worse, but I’ve found that because we do a Twitter chat, so we’re finding out stuff about what people want to know and it’s just keeping your brain active. If you just sat and watched the television, you’d go loopy, you know you’d go loopy. I found that keeping very active helps. (P16)

Indeed, many participants were thankful for the cognitive stimulation that digital technologies offered, with some feeling that it provided a protective mechanism against symptom progression. Prior research highlights the importance of cognitive stimulation for people with dementia (e.g. Aguirre et al., 2013). Our findings suggest that digital technologies may provide one way of facilitating cognitive stimulation, which may be particularly important during periods of lockdown when opportunities for stimulation are limited.

### Technologies to find meaning

Researchers have recommended that technologies should be designed to support the higher-level needs of people with dementia, facilitating activities that boost self-esteem and self-actualisation (Dixon & Lazar, 2020). Consistent with this, we found that participants reported using digital technologies to counter a loss of meaning and self-worth during the pandemic, when opportunities for self-actualisation were limited (Talbot & Briggs, 2021). Participants described using technologies for self-development, whereby they used sites such as YouTube or videoconferencing software to facilitate learning. For example, one participant discussed their experience of using YouTube to learn crafting skills:

I learned it through YouTube. I can’t see the patterns very well so - on YouTube they do new different patterns that you can do, and they do it from start to stop so it’s usually easier for me so I’ve made quite a few. (P18)

For others, learning how to use technologies was a new skill, which provided them with a sense of achievement. J1 spoke of using videoconferencing software for the first time:

Obviously for meetings, for Zoom and Microsoft, other sorts of things, so it is like learning a new skill. (P6)

Other participants reported using technologies to support their advocacy work. This involved using video-calling software, social media, smartphones, tablets or laptops to give talks about dementia; blog/vlog about lived experience and educate organisations. One participant said he used Zoom to advise a local hospital on how to be more dementia-friendly:

I’ve been acting as a liaison officer as a dementia advisor so that the hospital is dementia-friendly. And I’ve been down there once and when the restrictions took place, I’ve had a lot of Zoom meetings and team meetings with them. (P6)

Using technologies to facilitate advocacy is important, given that advocacy can be an important coping mechanism for people with dementia, providing a way of regaining purpose, respect and ‘fighting back’ against the disease (Weetch, O’Dwyer, & Clare, 2020). Thus, at a time when meaningful offline activities were cancelled, online advocacy provided a suitable alternative that facilitated self-worth and enabled some people to feel like they were continuing to contribute to society.

In addition to online advocacy, participants said they used technologies to enhance self-worth by taking part in research, acting as advisors on studies, helping others with dementia, continuing voluntary work and taking part in religious activities:

The wonders of technology - there’s voluntary work I do that involves people living with this condition at home. (P9)

Well church, I have church on Zoom and bible study on Zoom as well on a Tuesday morning which has helped me. (P3)

This use of technology engendered a sense of purpose among participants; as one participant stated, it made them ‘feel needed’. Meaningful activity is important for people with dementia as it can preserve dignity and a sense of identity (Roach & Drummond, 2014). Our findings suggest that digital technologies can enhance feelings of self-worth for some people with dementia, thereby enhancing personal experience and potentially helping them to ‘live well’ with the diagnosis.

### Hypercognitive technologies

People with dementia may face accessibility issues when using technologies, such as difficulties with readability and issues with navigation (Joddrell & Astell, 2016). In our interviews, we found that participants described cognitive fatigue and usability issues as the types of challenges they faced. Video-calling software was described as particularly cognitively demanding, with many participants expressing that they experienced ‘Zoom fatigue’:

I overused zoom to start with. On the day, my eyes were dry and tired, couldn’t concentrate. The day after would be a “foggy” day, meaning brain on a real go slow, could do nothing. (P8)

While Zoom fatigue has not been empirically tested or fully conceptualised in research, its effects appeared strong for people with dementia, causing headaches, fatigue and enhanced problems with concentration. This may be because conversations via video-calling software were more demanding for participants. For example, participants described the challenge of managing conversations without non-verbal cues:

The face to face I can sort of - not lip read - but sort of know what they’re saying but it’s still difficult, a lot more than a normal meeting because well sometimes it can go that way in a call. (P18)

Non-verbal cues such as bodily contact, eye contact and gestures can promote effective communication with people with dementia (Jootun & McGhee, 2011). However, our findings suggest that some of these cues may not translate well into online settings, thus inhibiting communication.

Participants also described perceptual problems, whereby they found it difficult to process images or recognise others on screens. This gave them headaches, created awkward situations and exacerbated feelings of embarrassment and exclusion:

A lot of Alzheimer’s stuff really, recognizing people, and when I get on the Zoom, I can get embarrassed. So, I just come out of it because I can’t put their names up on the screen and can’t remember who they were from last week, so that’s upsetting really because I don’t feel part of the group. (P7)

Another key challenge concerned participants’ ability to navigate and remember how to use digital technologies. As a result, participants often felt they were reliant on others:

I eventually got around to using technology but I have to have someone on hand or someone to set it up for me unless I’ve done something wrong with technology, I press the wrong button. (P7)

I wouldn’t be able to do it if my husband wasn’t here. (P1)

While assistance from family members was necessary, participants expressed that they wanted to use technologies independently. They provided suggestions for promoting independent technology usage among people with dementia, including training courses and dementia-friendly instructions. One participant suggested that accessible instruction cards could be sent to people with dementia:

Maybe there can be a sort of card that can be sent, when you’re setting up a Zoom meeting, maybe a card can be sent through, or an email, with it on there. (P1)

This could be beneficial for people with dementia, promoting their independence by enabling them to use technologies without relying on others. In turn, this may promote self-growth and enhance levels of mastery.

## Discussion

We examined how people with mild-to-moderate dementia used digital technologies during the COVID-19 pandemic to examine the benefits and challenges these technologies presented. Using a positive technology lens, we found that digital technologies such as video-calling software and social media hold great potential to boost social connectedness, well-being and self-actualisation among people with dementia. In line with our critical dementia approach, we recognise that such technologies helped many participants to maintain a sense of self as a contributor to society, whether through digital volunteering or through skills acquisition (cf Brankaert et al., 2019). Our findings suggest that the people with dementia who participated in this study cannot be viewed as passive actors who experienced the pandemic stoically. Instead, their statements suggest they are active agents who actively sought out technologies to meet their needs, which contrasts with a public dialogue that often identifies them as ‘victims’ and ‘sufferers’ (Peel, 2014). Thus, it is important that we continue to move beyond approaches that infantilise and disempower people with dementia and recognise them as people with agency.

Despite these benefits, participants also outlined the challenges they faced when using technologies. For example, they found it cognitively demanding, experienced perceptual difficulties and struggled to manage online conversations. In some cases, even when technologies were available that could assist with everyday life (i.e. online shopping), these were ineffective as policies did not identify people with dementia as ‘vulnerable’. As there is increasing pressure for offline spaces to be dementia-friendly (e.g. Alzheimer’s Society, 2013), we advocate that online spaces should also be accessible for and inclusive of people with dementia. Designers could therefore consider engaging people with dementia throughout all stages of the design process, thereby adopting a ‘nothing about us without us’ approach to HCI.

While participants demonstrated an ability to learn technological skills, they often expressed a need for training on how to use digital technologies. This training could be particularly helpful for people with dementia who live alone or who may not have support directly available to them. We therefore recommend such training is developed and helpful resources are made available. As one participant suggested, dementia-friendly leaflets could be developed that contain accessible information on how to use technologies, such as email, Twitter and Zoom. However, it is important that resources are co-produced with people with dementia. Other researchers have developed initiatives that aim to support older adults in training their peers to be aware of cybersecurity threats and how to counter them (e.g. CyberGuardians; Nicholson & McGlasson, 2020). A similar co-designed programme on how to use digital technologies may be beneficial for people with early-stage dementia. This may serve to promote digital engagement, literacy and inclusion among people with dementia, as well as fostering a sense of community. In turn, this may enhance well-being, combat isolation and improve self-worth.

### Limitations

Despite our promising findings, there are some limitations of our work. Firstly, we did not include operationalised measures of dementia, well-being or social connectedness in the study design and instead relied on self-reports. In the future, such measures could be included to verify diagnoses and further explore associations between technology usage and well-being.

Participants were recruited from social media and remotely through dementia networks. Therefore, our sample may have been more likely to have positive views of technology. Consistent with this, the participants in our sample often recognised that they occupy privileged positions to be able to access the internet and digital technologies. This may not be the case for individuals who live rurally or who are from lower socio-economic backgrounds. In the future, researchers could speak to people with dementia from diverse backgrounds and individuals who do not use technology to examine the barriers they face.

Our sample was also relatively young, with most participants having young-onset, mild-to-moderate dementia. Research suggests that people with dementia who use technologies such as social media tend to be relatively young (Talbot et al., 2020a), which begs the question of whether our findings in relation to agency and learning are limited to this group. Of course, there are likely to be cohort effects here, such that digital competence will improve as younger generations age, but digital competence may also decline as dementia progresses. That being said, with careful design, even those with more severe dementia and with relatively little digital experience can develop digital agency, for example, in a digital art setting, as demonstrated by Lazar et al. (2017). Therefore, we remain positive about this approach and argue that our study provides an important foundation for understanding how people with dementia use digital technologies during a time of change and the challenges they face.

## Conclusion

In conclusion, people with mild-to-moderate dementia used digital technologies to facilitate social connection, improve well-being, promote self-actualisation and assist with everyday life during the COVID-19 pandemic. For example, they used video-calling software to access peer support and combat isolation; games and social media for cognitive stimulation and YouTube videos to learn new skills. Participants viewed these technologies as vital in combatting the social isolation, loss of self-worth and lack of stimulation that accompanied the pandemic. Therefore, our findings speak to the ability and willingness of participants to adapt to the stresses of the pandemic and use technology to meet their needs, suggesting that they should be viewed as active agents rather than passive users. Despite these benefits, people with dementia sometimes found it difficult to use technologies, experiencing cognitive fatigue and facing usability issues. In some cases, even when digital technologies were available and could be used to assist with everyday life, these were ineffective due to policies that did not identify people with dementia as ‘vulnerable’ and therefore provided a barrier to accessing basic necessities. In future, we recommend that training on how to use technologies is co-produced with people with dementia and that designers of digital technologies engage with the voices of people with dementia throughout the design process. In turn, this could promote the digital inclusion, social connectedness, well-being and self-worth of people with dementia.
